# Indirect Single-Step Apexification Utilizing a Carrier-Supported Custom-Fit Biomaterial: A Novel Technique Using Mineral Trioxide Aggregate and Biodentine

**DOI:** 10.7759/cureus.115720

**Published:** 2026-09-03

**Authors:** Damini Kesarwani, Sunandan Mittal, Vanita Keshav, Tarun Kumar

**Affiliations:** 1 Conservative Dentistry and Endodontics, Dasmesh Institute of Research and Dental Sciences, Faridkot, IND

**Keywords:** apexification, apical barrier, biodentine, custom-fit biomaterial, endodontics, mineral trioxide aggregate, open apex, root canal treatment

## Abstract

Management of teeth with open apices remains technically challenging because conventional apexification techniques may be associated with prolonged treatment, multiple clinical visits, difficulty in controlling apical barrier placement, and the risk of extrusion of biomaterial beyond the apical foramen. This technical report involves capturing the three-dimensional internal morphology of the prepared root canal using a light-body silicone impression, reproducing the canal configuration in heavy-body putty, fabricating a mineral trioxide aggregate (MTA) or Biodentine plug within the negative replica, and incorporating a twisted stainless-steel ligature wire to facilitate retrieval and controlled intracanal placement. The technique was clinically applied to two teeth with open apices and periapical lesions. Biodentine was used in a 32-year-old woman with an open apex in the maxillary left central incisor, whereas MTA was used in a 45-year-old man with an open apex in the mandibular right first premolar. Both cases showed favorable clinical and radiographic outcomes during 24 months of follow-up. The Periapical Index score improved from 4 to 1 in both cases, and no apical extrusion of the biomaterial was observed. The described technique suggests the feasibility of indirect fabrication and controlled placement of a custom-fit apical biomaterial plug. Further laboratory and clinical studies are required to evaluate its dimensional accuracy, sealing ability, mechanical behavior, biological response, and long-term clinical performance.

## Introduction

An open apex is characterized by incomplete root development, resulting in a wide apical foramen and the absence of a conventional apical constriction [1]. Interruption of root development may occur following trauma or carious pulpal exposure with subsequent pulp necrosis and cessation of dentinogenesis [2]. Other reported etiologic factors include severe apical or periapical resorption and iatrogenic enlargement of the apical foramen [3].

Apexification is traditionally performed to establish an artificial apical barrier that facilitates subsequent root canal obturation in teeth with necrotic pulps and open apices [4]. Conventional calcium hydroxide apexification may require prolonged treatment over several months and multiple appointments and has been associated with weakening of the root structure and a risk of reinfection during the treatment period [5]. The introduction of bioactive calcium-silicate materials, including mineral trioxide aggregate (MTA) and Biodentine, has facilitated direct placement of an apical barrier rather than waiting for the biological formation of an apical barrier, as with traditional calcium hydroxide apexification, thus expanding the treatment options for immature teeth [6,7].

Despite these developments, direct orthograde placement of a hydraulic calcium-silicate biomaterial into a wide or irregular open apex remains technique-sensitive. The clinician must control the quantity and position of the material while avoiding extrusion into the periapical tissues [8,9]. Accurate adaptation of a manually placed apical plug may also be difficult in cases with a large, irregular, or anatomically complex apical opening.

The proposed technical report describes an indirect approach in which the three-dimensional internal canal morphology is captured using an elastomeric impression and subsequently reproduced to fabricate a patient-specific apical biomaterial plug. By allowing the apical barrier to be customized before clinical placement, this approach may facilitate more controlled positioning in anatomically complex open-apex canals. This technique was clinically applied to two teeth with open apices, with clinical and radiographic follow-up over 24 months. The technique is currently under consideration for further clinical and radiographic evaluation.

## Technical report

Technical rationale

The proposed technique is based on the concept that an individualized biomaterial plug can be fabricated outside the root canal after recording the three-dimensional morphology of the prepared canal. Instead of incrementally placing hydraulic biomaterial directly into the open apex, the canal anatomy is first reproduced in an impression and then transferred to a negative replica mold. The biomaterial is subsequently packed into this replica to produce a plug corresponding to the recorded canal geometry.

The incorporation of a twisted stainless-steel ligature wire provides a means of retrieving the partially set plug from the mold and subsequently carrying it into the root canal. The wire also provides structural support during manipulation. The technique therefore separates plug fabrication from clinical placement and allows inspection of the fabricated plug before its definitive insertion.

Materials

The biomaterials used were Biodentine (Septodont, Saint-Maur-des-Fossés, France) in Case 1 and MTA (Angelus, Londrina, PR, Brazil) in Case 2. The bioactive sealers were BioRoot Flow (Septodont, Saint-Maur-des-Fossés, France) for Case 1 and MTA Fillapex (Angelus) for Case 2.

The impression materials consisted of light-body and heavy-body putty silicone (Flexceed, GC Corporation, Tokyo, Japan). A twisted stainless-steel ligature wire was used as the carrier. The working length was determined using an electronic apex locator (Root ZX II, J. Morita, Kyoto, Japan) and verified by digital radiography.

Technique steps

Step 1: Working Length Determination

Following standard access cavity preparation, the working length was determined using an electronic apex locator and verified radiographically (Figures 1, 2). The working length was established before preparation of the canal impression.

**Figure 1:**
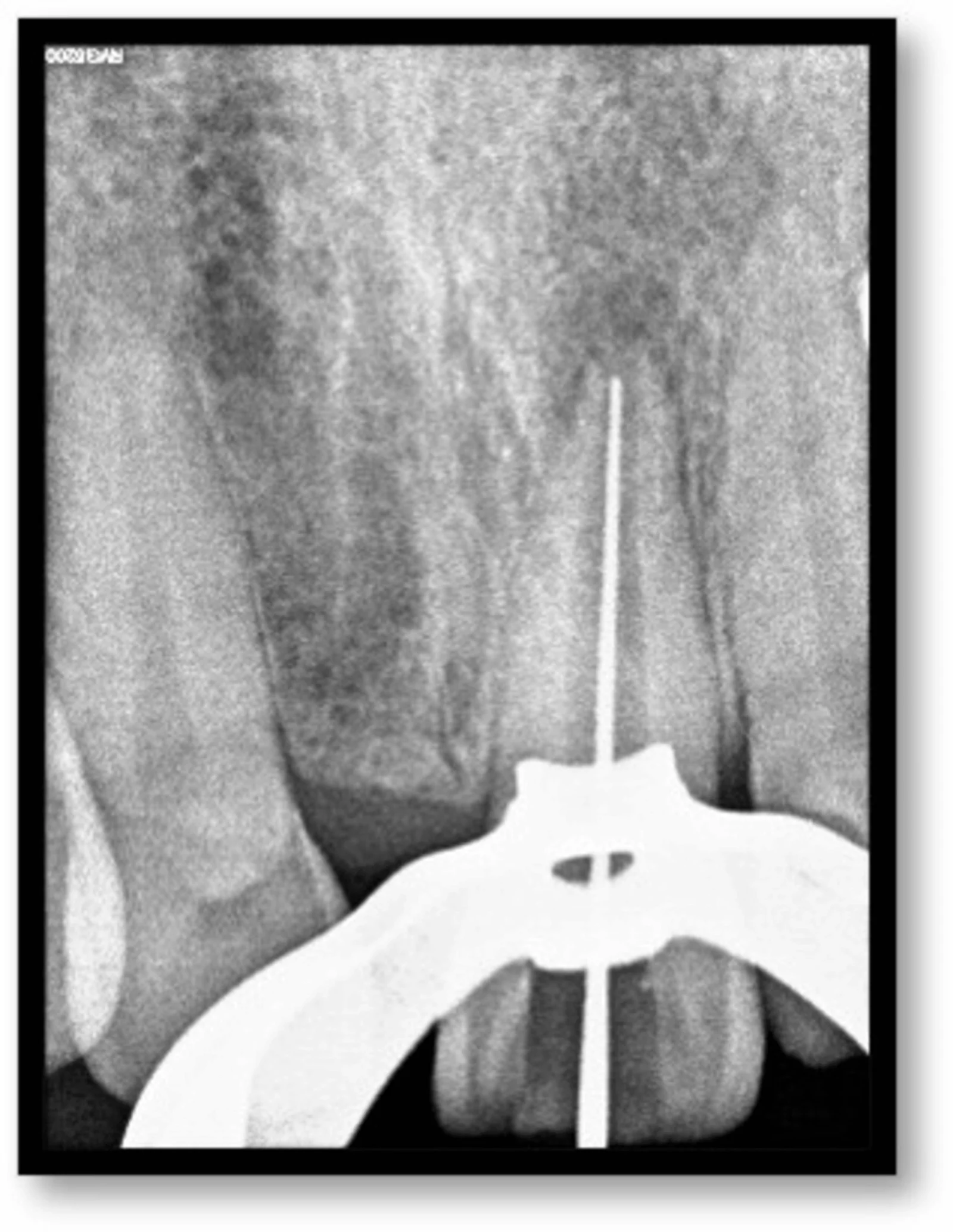
Working length determination for Case 1

**Figure 2:**
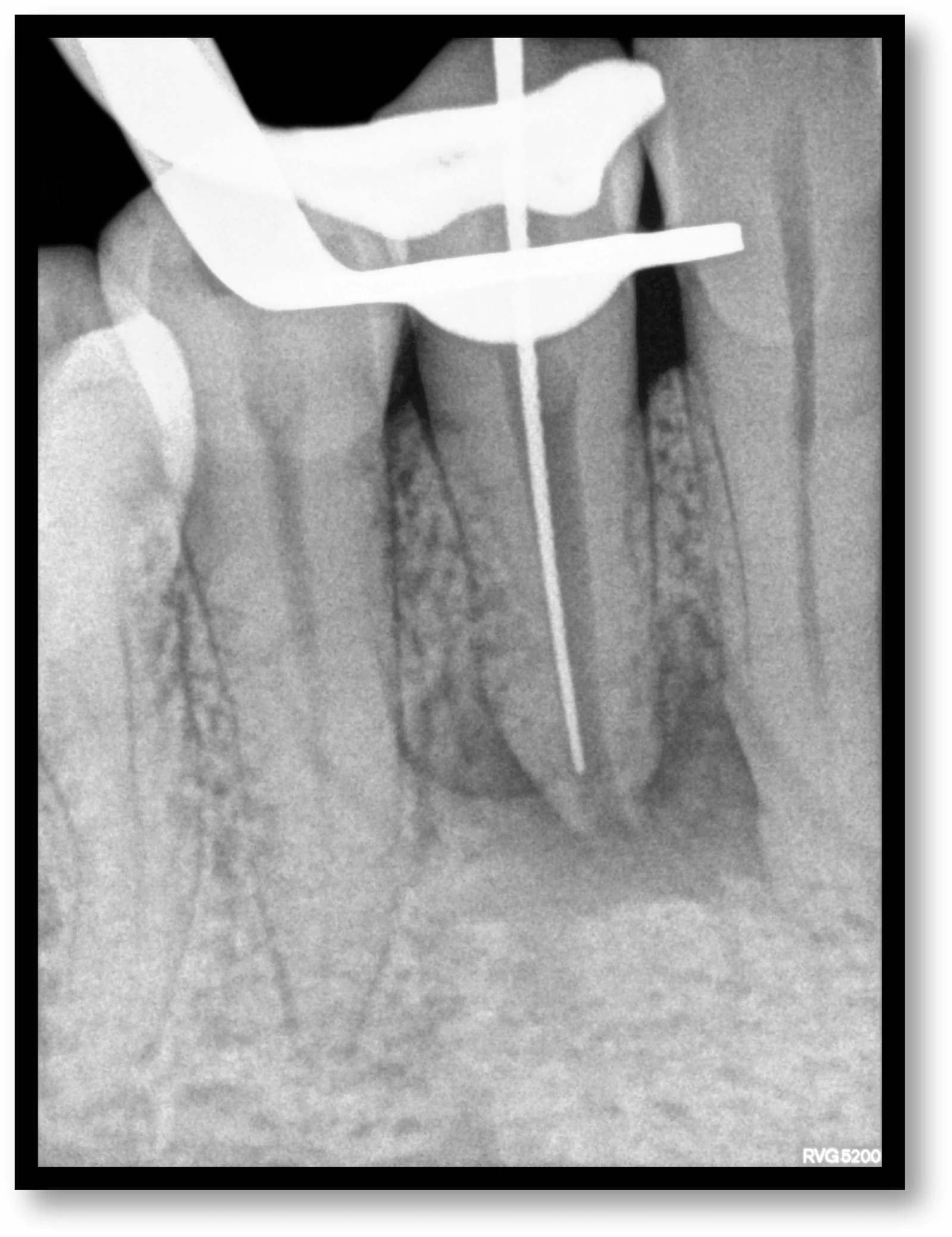
Working length determination for Case 2

Step 2: Canal Preparation and Intracanal Impression

Biomechanical preparation was performed using 3% sodium hypochlorite and 17% EDTA. A triple antibiotic paste (TAP), consisting of ciprofloxacin, metronidazole, and minocycline in equal proportions (1:1:1), was prepared using sterile saline as the vehicle and placed as an intracanal medicament for two weeks. TAP was used to reduce the intracanal microbial load and facilitate adequate disinfection prior to the subsequent apexification procedure.

At the subsequent appointment, the intracanal medicament was removed, and the canal was irrigated and dried with sterile paper points. In the present cases, the canals had open apices, and no conventional apical constriction was present. Therefore, the predetermined working length served as the controlled apical limit for introduction of the impression material. A light-body silicone impression was introduced into the canal to the predetermined working length. To ensure precision and prevent material extrusion, a wooden toothpick coated with light-body impression material was used as a carrier and clearly marked at the predetermined working length. The impression was allowed to polymerize for approximately three minutes and was then carefully retrieved and inspected under 3.5× magnification for continuity and completeness. The retrieved impression was also assessed for any evidence of extension beyond the intended apical limit; no clinically apparent extrusion was observed (Figures 3, 4). 

**Figure 3:**
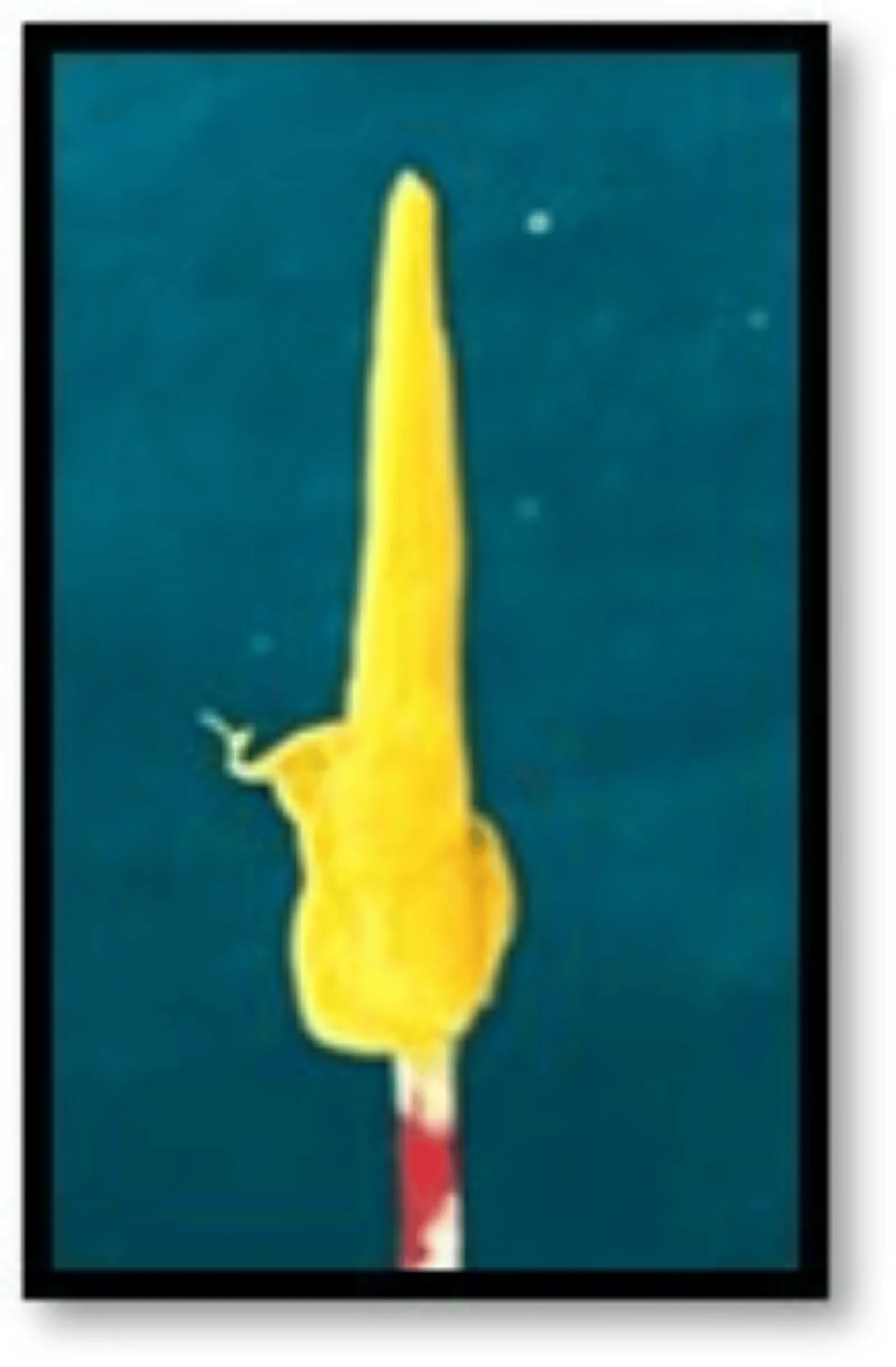
Retrieved light-body silicone intracanal impression under 3.5× magnification, capturing the three-dimensional internal canal anatomy for Case 1

**Figure 4:**
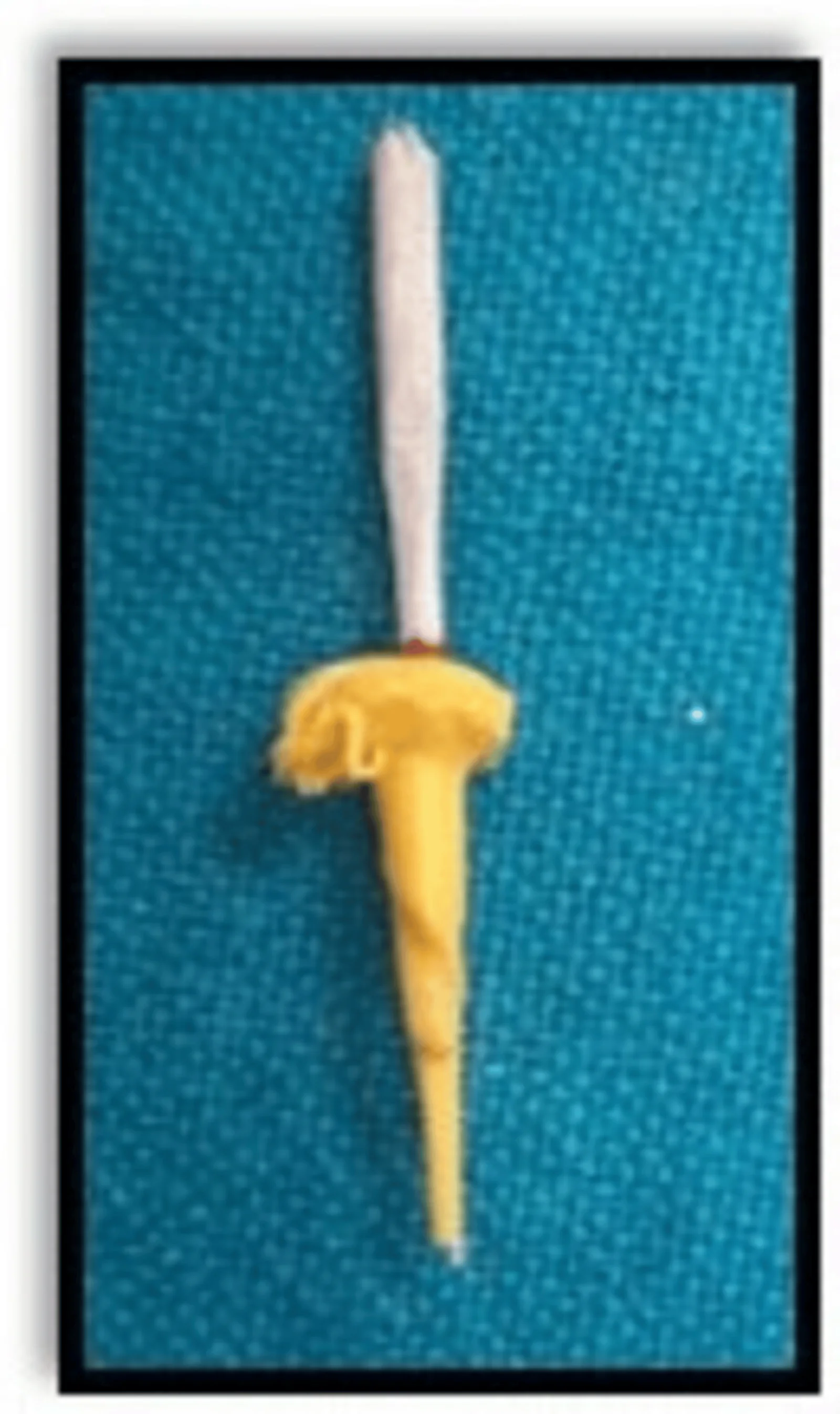
Retrieved light-body silicone intracanal impression under 3.5× magnification, capturing the three-dimensional internal canal anatomy for Case 2

The purpose of this step was to record the internal morphology of the prepared root canal, including the apical configuration, before fabrication of the custom-fit biomaterial plug.

Step 3: Negative Replica Fabrication

The completed light-body silicone impression was positioned within heavy-body putty silicone (Figure 5). The putty was allowed to set for approximately five minutes, thereby creating a negative replica corresponding to the shape and length of the recorded canal.

**Figure 5:**
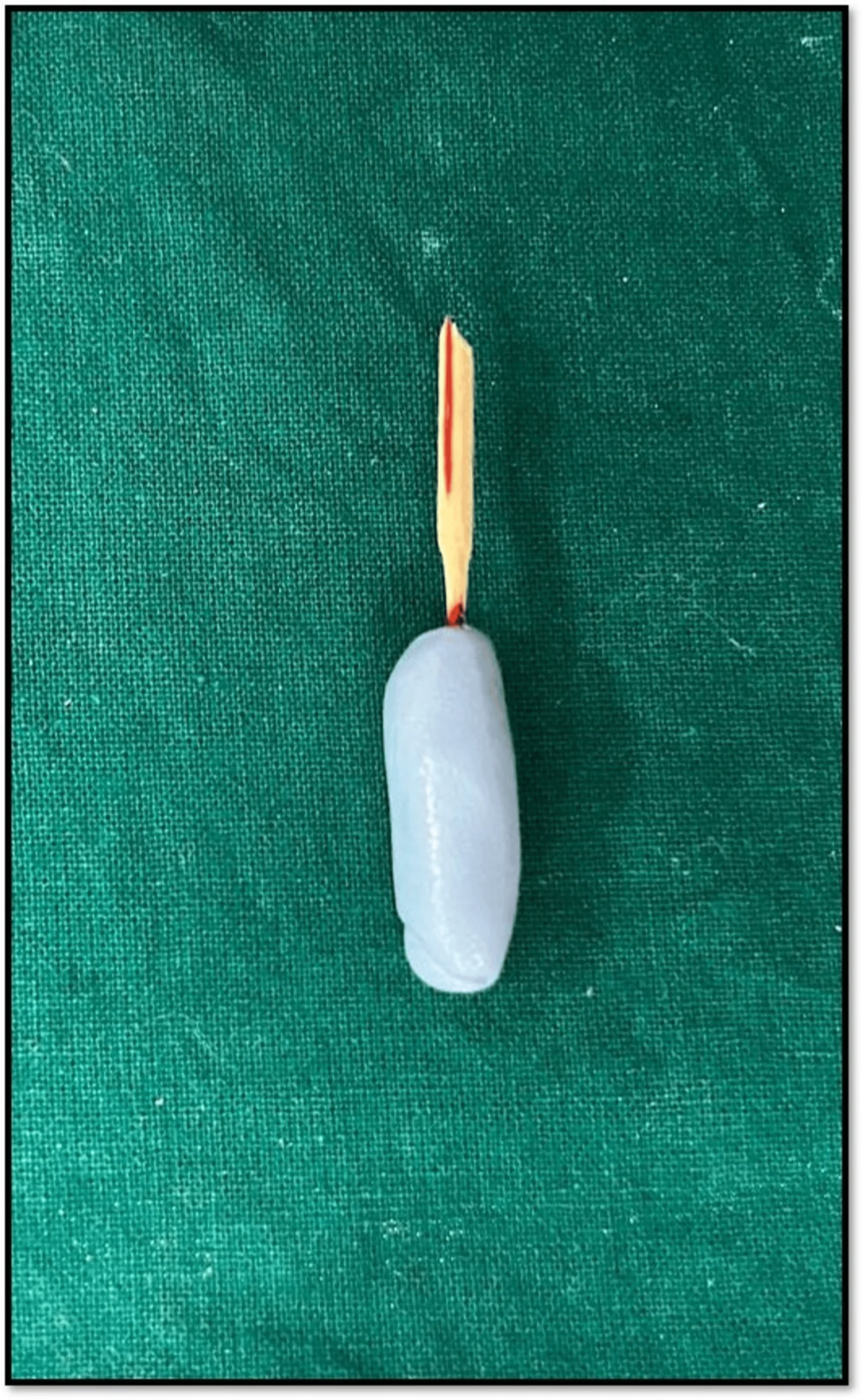
The light-body silicone impression positioned within heavy-body putty silicone

Step 4: Mold Preparation

After complete setting of the heavy-body putty, the mold was carefully divided into two halves using a scalpel blade. The light-body silicone impression was removed (Figure 6). Splitting the mold allowed access for biomaterial placement and facilitated subsequent retrieval of the fabricated custom-fit plug. 

**Figure 6:**
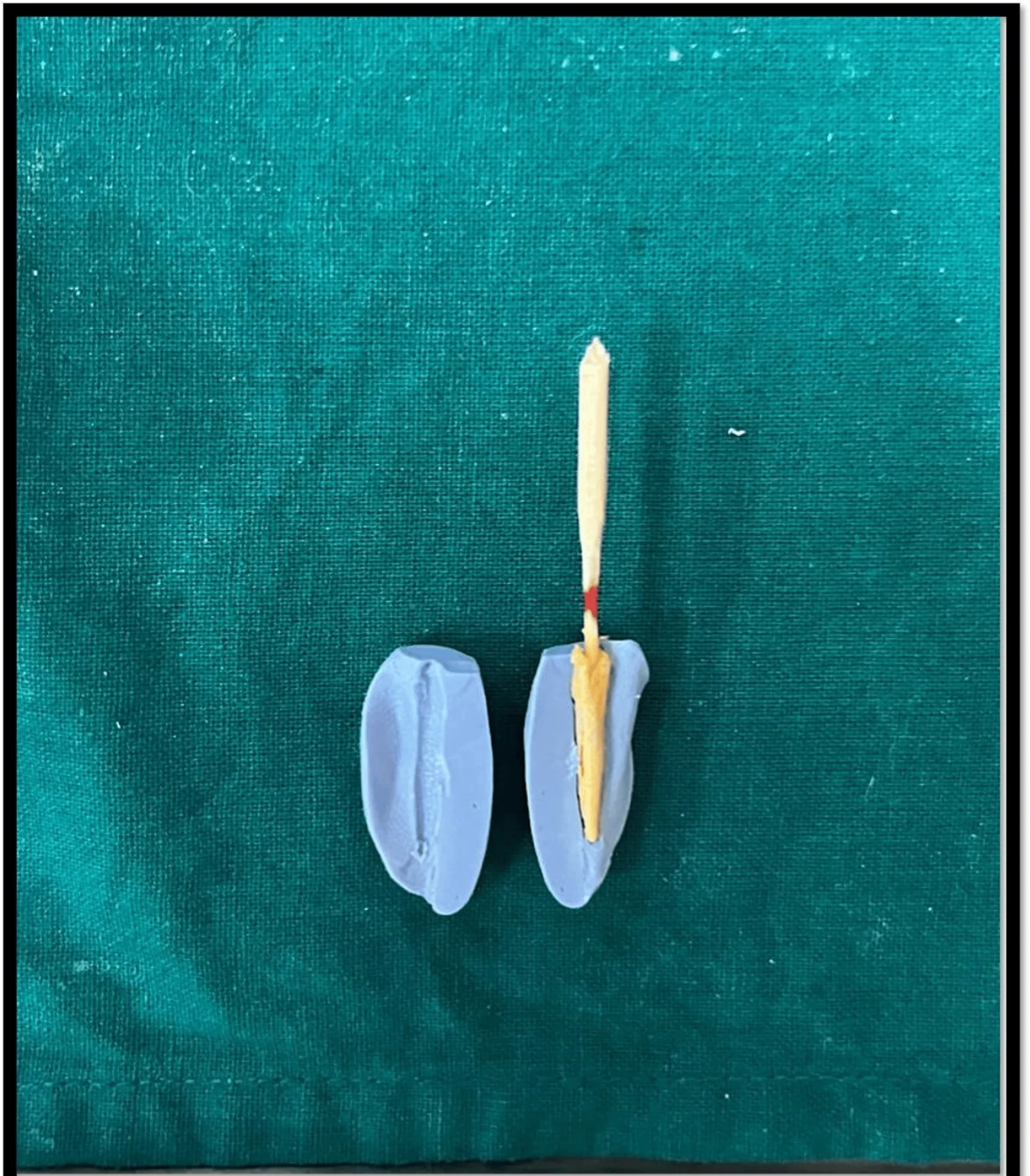
Split putty mold

Step 5: Custom Biomaterial Fabrication

MTA or Biodentine was mixed according to the respective manufacturer's instructions. The freshly mixed biomaterial was incrementally packed into the negative replica using a condenser.

Before initial setting, a stainless-steel ligature wire measuring 0.2 mm in diameter was twisted to produce a final diameter of approximately 0.5 mm. The twisted wire was centrally incorporated along the longitudinal axis of the customized plug, with its total length maintained approximately 2-3 mm shorter than the plug. This ensured complete encapsulation of the wire within the biomaterial without exposure at the apical end (Figure 7). 

**Figure 7:**
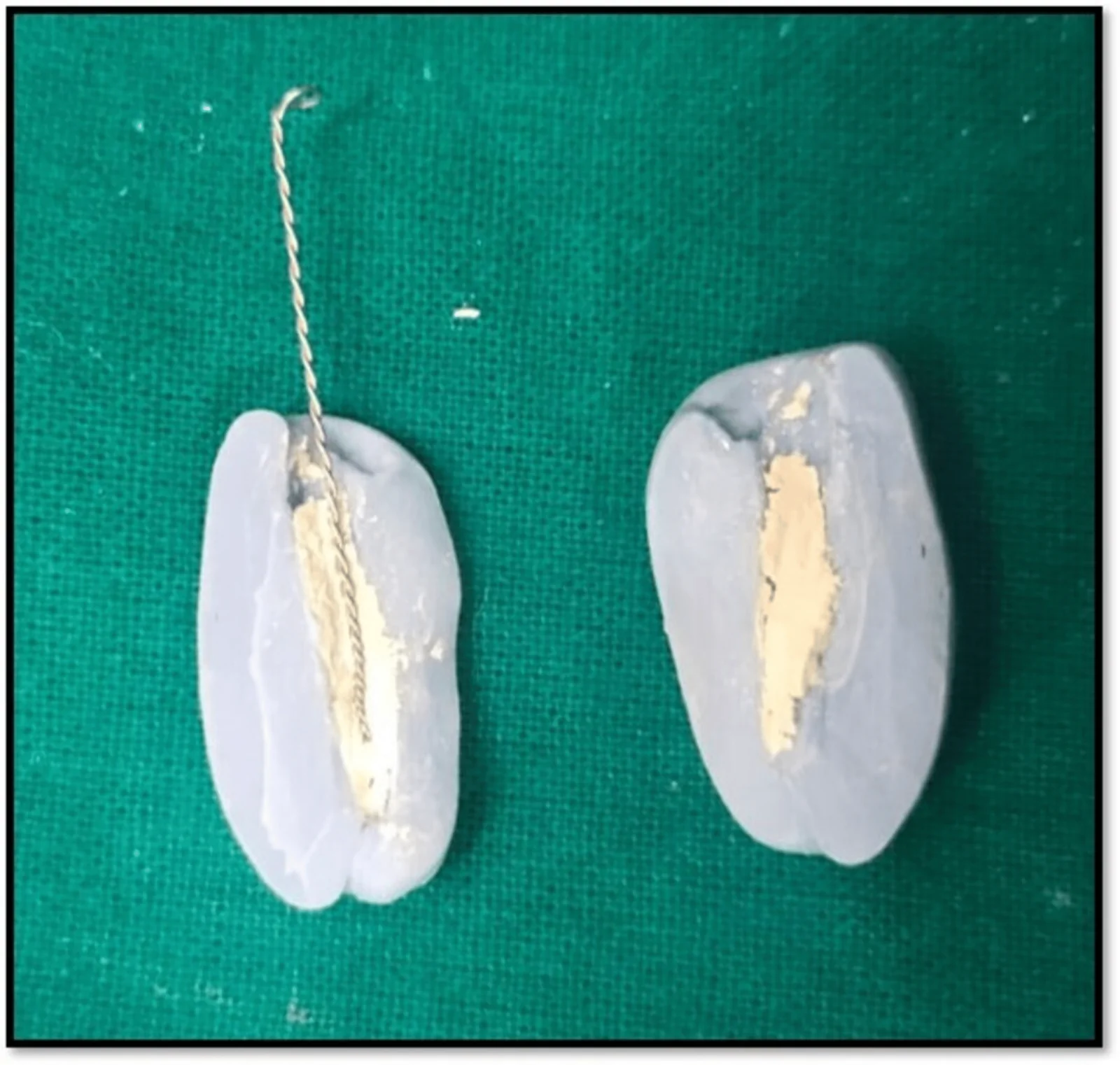
Split putty mold showing biomaterial packing with embedded twisted ligature wire

The embedded wire was incorporated primarily for mechanical reinforcement and controlled manipulation of the customized biomaterial plug during insertion and retrieval. It also provided structural support during handling. The assembly was allowed to reach the initial setting within the mold. According to the manufacturer's guidelines, the initial setting times are approximately 15 minutes for MTA and 12 minutes for Biodentine. 

Step 6: Retrieval and Verification

After initial setting, the custom-fit biomaterial plug was retrieved from the split putty mold using the embedded ligature wire. The fabricated plug demonstrated a tapered configuration corresponding to the recorded canal anatomy (Figure 8). 

**Figure 8:**
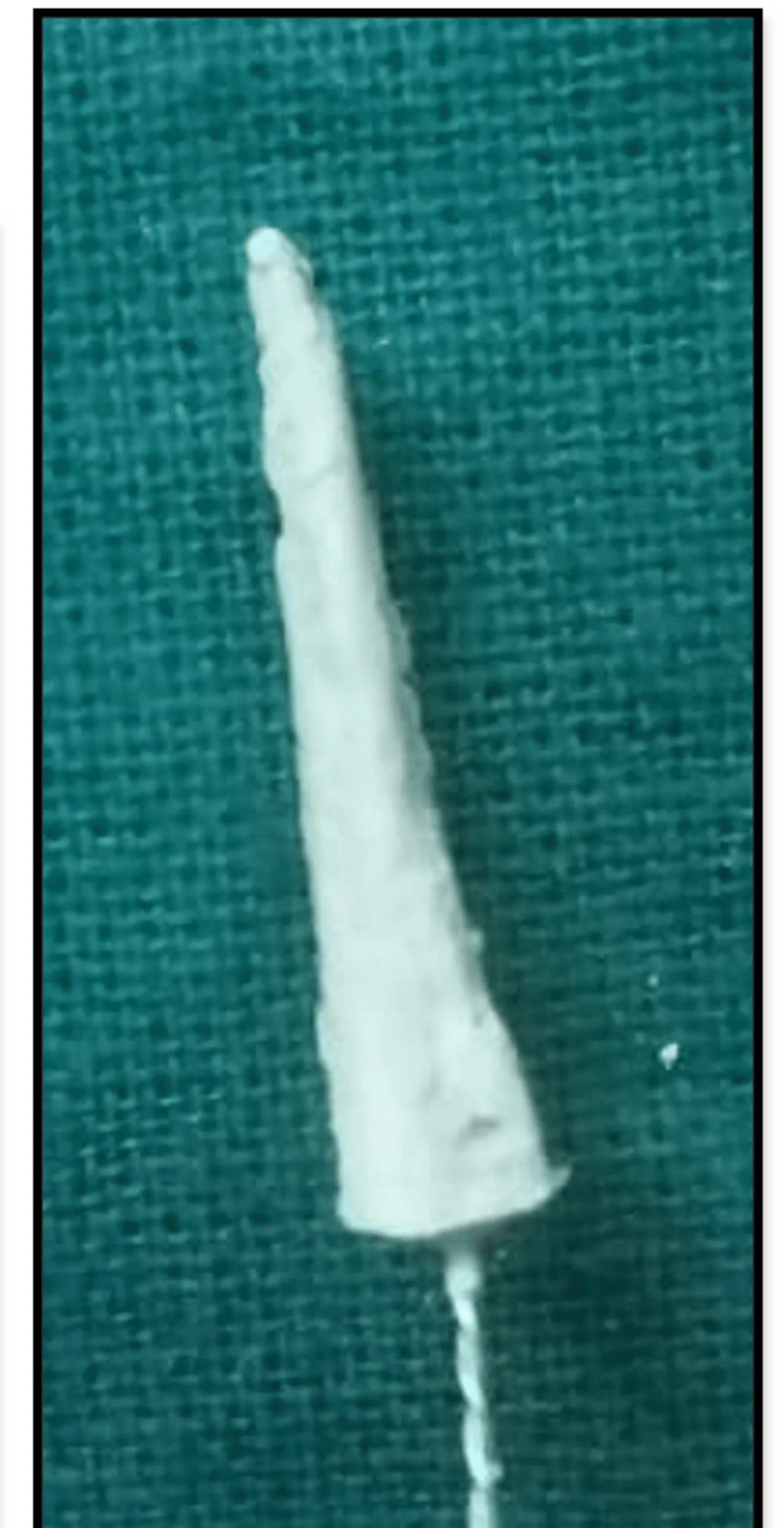
The fabricated custom-fit biomaterial plug under 3.5x magnification

The plug was inspected under 3.5× magnification for completeness and structural integrity. Its position and apicocoronal extent within the canal were subsequently verified radiographically before definitive placement.

Step 7: Clinical Placement and Restoration

A thin layer of bioactive sealer was applied to the canal walls using a Lentulo spiral at 300 rpm. BioRoot Flow was used in Case 1 and MTA Fillapex in Case 2. The thin sealer layer was used as an interfacial adjunct to minimize interference with seating of the customized plug while providing an additional interfacial sealing effect, with the potential for the sealer to penetrate into the dentinal tubules, thereby contributing to a continuous, monoblock-like sealing interface between the dentinal walls and the biomaterial plug.

The custom-fit plug was then positioned to the predetermined working length using gentle apical pressure. Once the plug was seated, the excess ligature wire was trimmed above the canal orifice. The access cavity was subsequently sealed with composite resin to reduce the potential for coronal microleakage (Figures 9, 10). 

**Figure 9:**
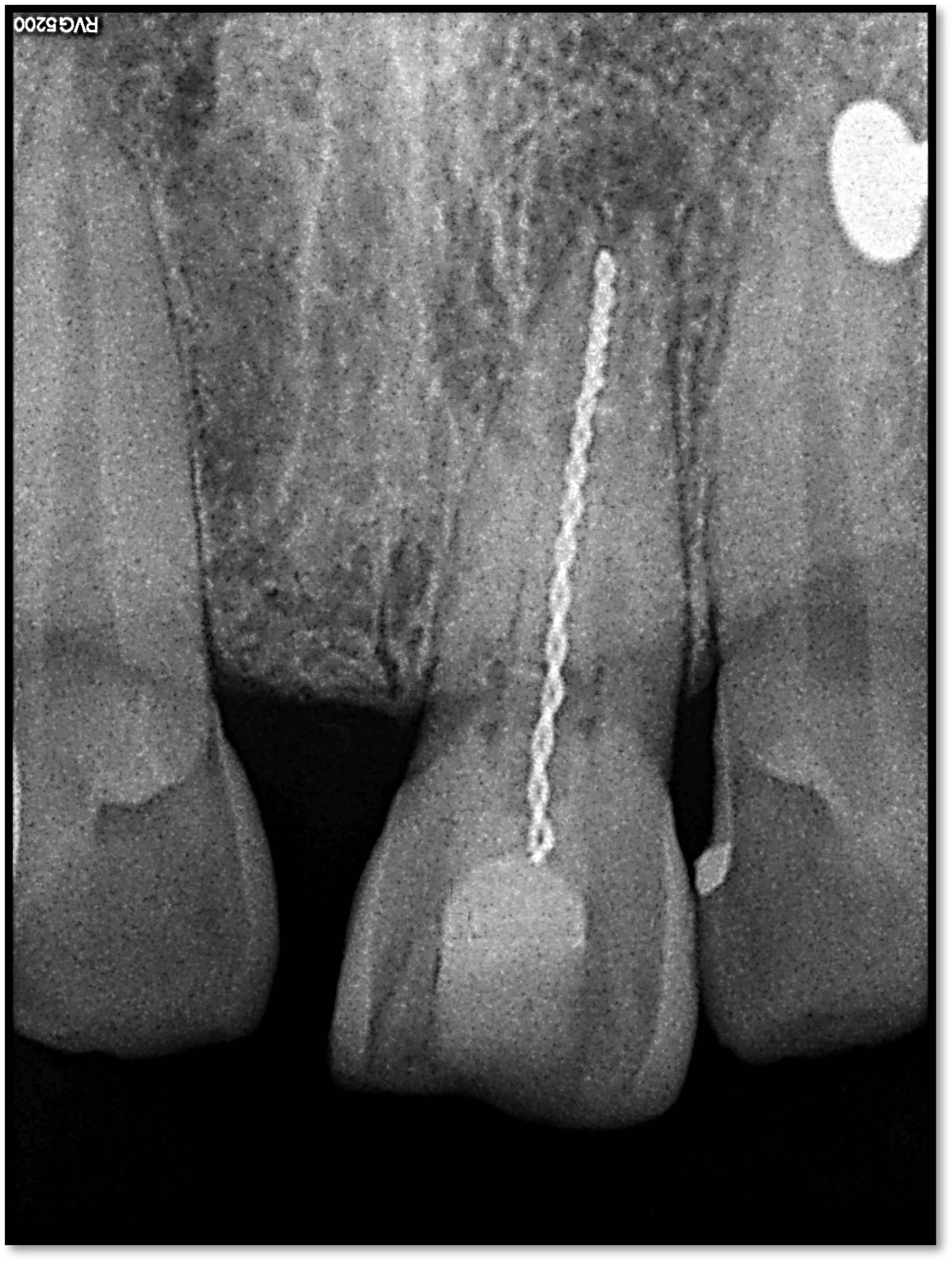
Postoperative radiograph (Case 1) following custom-fit biomaterial (Biodentine) cementation and post-obturation restoration

**Figure 10:**
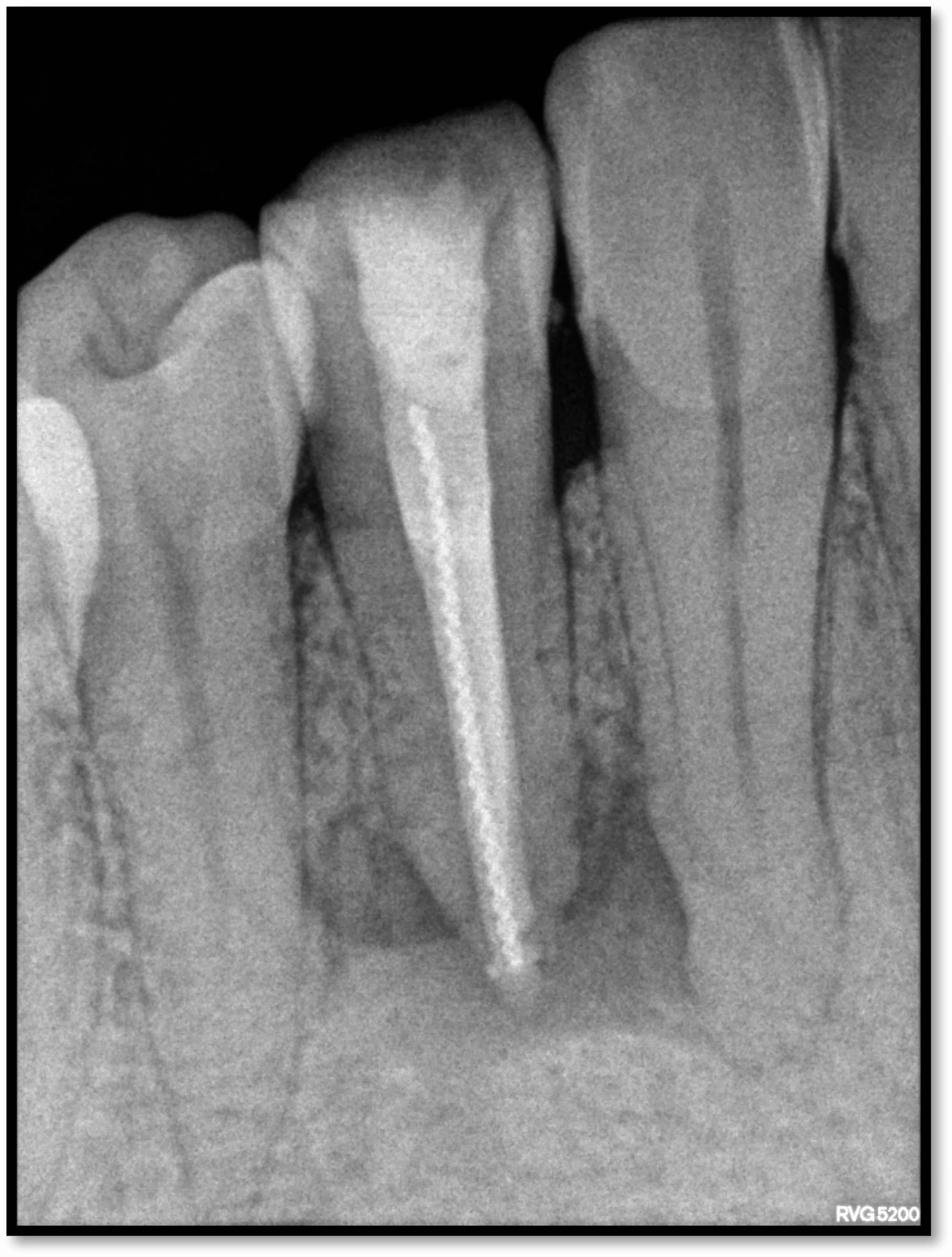
Postoperative radiograph (Case 2) following custom-fit biomaterial (mineral trioxide aggregate) cementation and post-obturation restoration

Clinical application

Case 1: Biodentine

A 32-year-old woman presented with discoloration of the upper anterior tooth and occasional pain on biting for six months. The patient reported a history of childhood trauma with loss of one central incisor. The reported biting discomfort was not specifically localized to tooth #21 and could not be reproduced during clinical examination.

Clinical examination demonstrated slight discoloration of the maxillary left central incisor (#21). Palpation and percussion were negative. Mobility was Grade 0, and periodontal probing depths were within physiological limits (≤3 mm).

Radiographic examination demonstrated an open apex associated with a periapical radiolucency (Figure 11). The periapical status was assessed using the five-point Periapical Index (PAI) scoring system described by Ørstavik et al. The radiograph was evaluated by the treating clinician according to the defined PAI criteria and assigned a PAI score of 4. Cold and electric pulp testing produced negative responses, consistent with pulp necrosis and asymptomatic apical periodontitis. 

**Figure 11:**
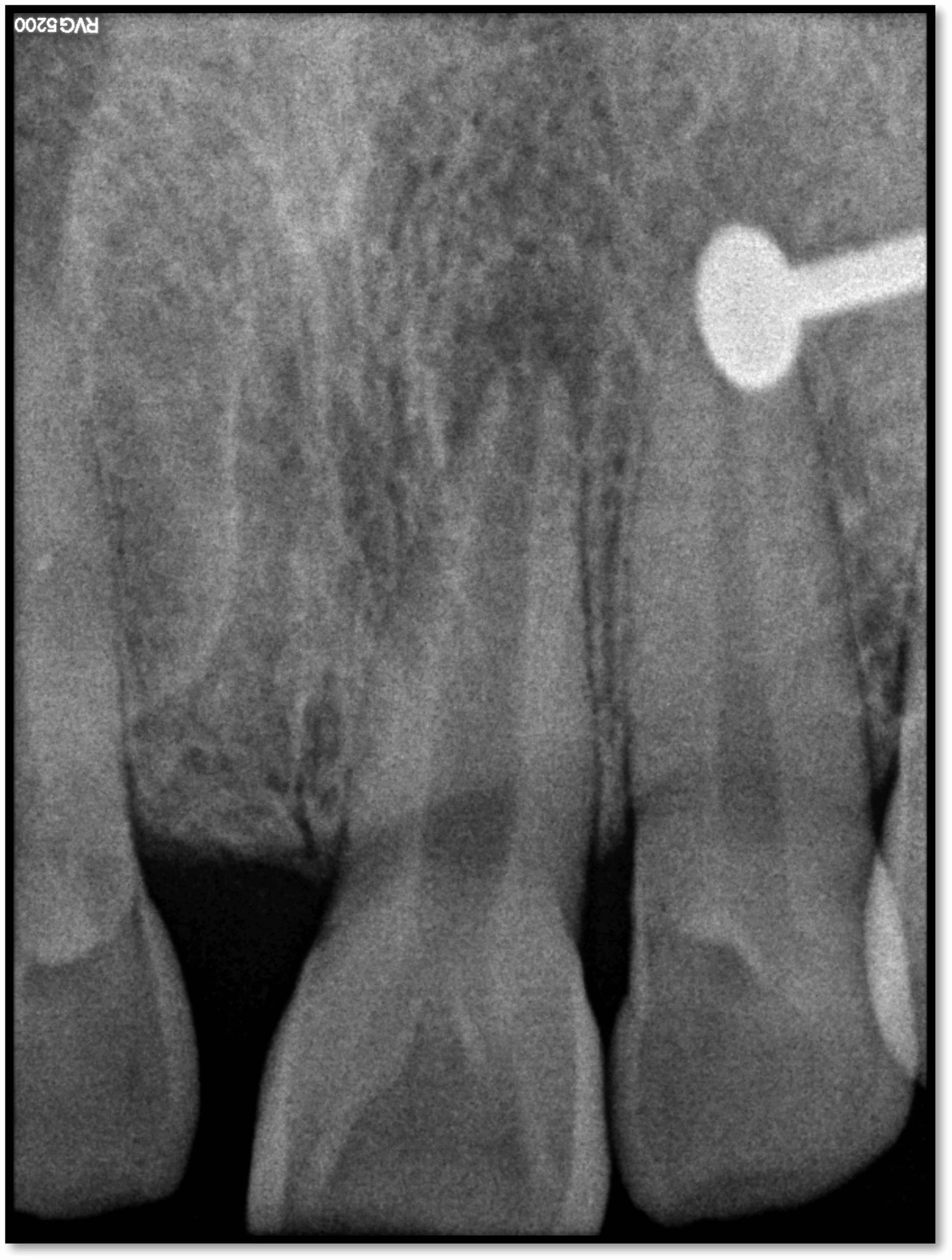
Preoperative radiograph showing open apex with periapical radiolucency (Case 1: Tooth #21, Biodentine)

The tooth was isolated using a rubber dam, and an access opening was made. The working length was established at 19 mm using a #40 K-file and digital radiography. Minimal circumferential filing was performed with copious irrigation using 3% sodium hypochlorite. The canal was dried, and triple antibiotic paste containing ciprofloxacin, metronidazole, and minocycline in a 1:1:1 ratio was placed as an intracanal medicament.

At the two-week appointment, the patient was asymptomatic. The intracanal medicament was removed using instrumentation and irrigation with 3% sodium hypochlorite and 17% EDTA. After drying, a light-body silicone impression was obtained. The custom-fit biomaterial plug was subsequently fabricated according to the described technique using Biodentine.

Case 2: Mineral Trioxide Aggregate

A 45-year-old man presented with moderate to severe continuous pain in the right mandibular posterior region for one week. The pain was alleviated with analgesics. There was no reported history of trauma.

Clinical examination showed that the mandibular right first premolar (#44) was positive on percussion and palpation. No intraoral or extraoral swelling was present. Mobility was Grade 0, and periodontal probing depths were within normal limits (≤3 mm).

Radiographic examination revealed an open apex with an associated periapical radiolucency (Figure 12). The PAI score was 4. Cold and electric pulp tests were negative. The diagnosis was pulpal necrosis with an open apex and symptomatic apical periodontitis. MTA was selected as the biomaterial for fabrication of the custom-fit apical plug. 

**Figure 12:**
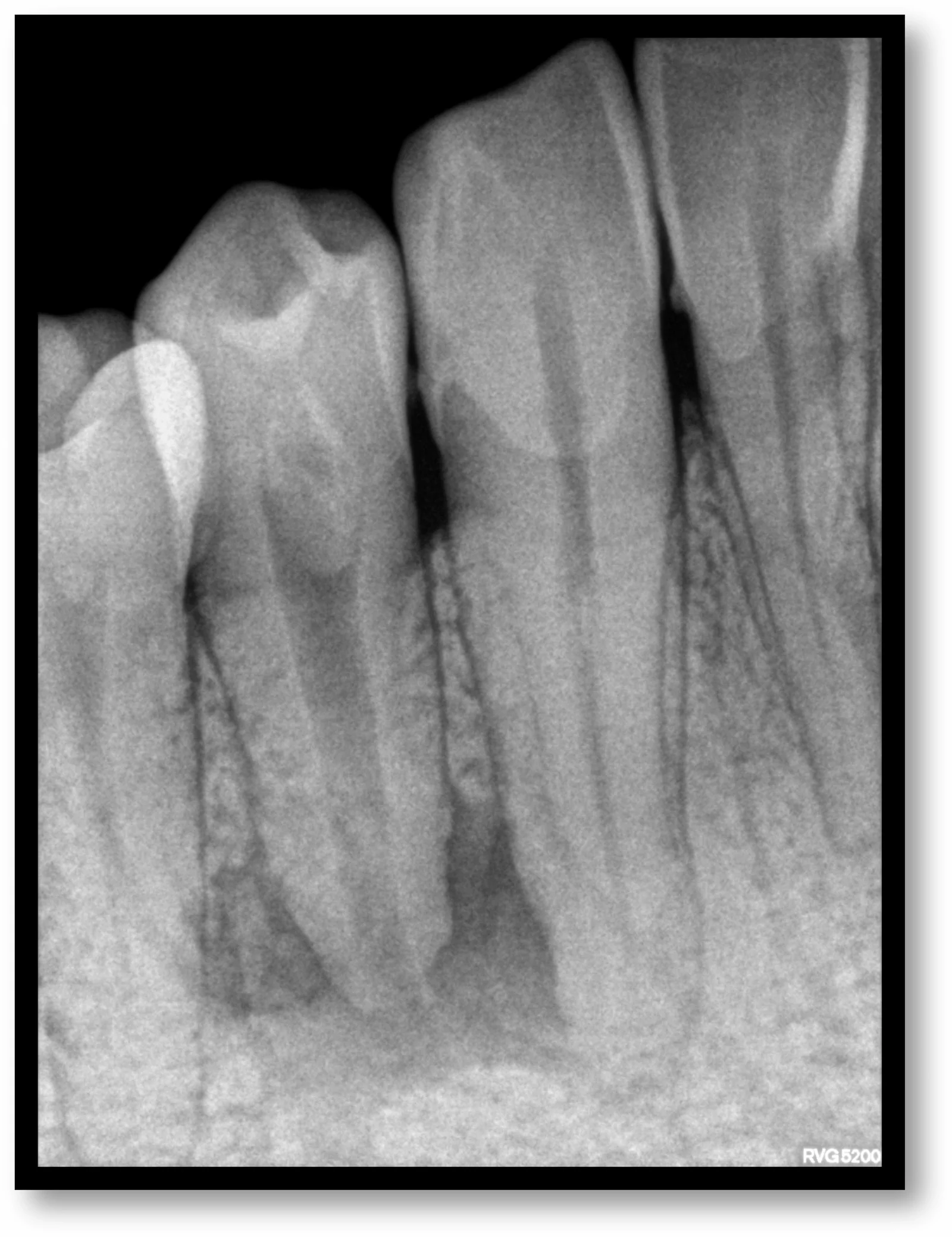
Preoperative radiograph showing open apex with periapical radiolucency (Case 2: Tooth #44, mineral trioxide aggregate)

Working length was established at 18 mm. The same clinical procedure was followed as in Case 1, and the intracanal impression was obtained according to the described technique. The custom-fit MTA plug was fabricated using the negative replica and embedded wire carrier and was subsequently inserted into the prepared canal.

Follow-up and Clinical Outcomes

Both patients were followed at three-month intervals during the first year and at six-month intervals thereafter, resulting in a total follow-up period of 24 months (Figures 13, 14). 

**Figure 13:**
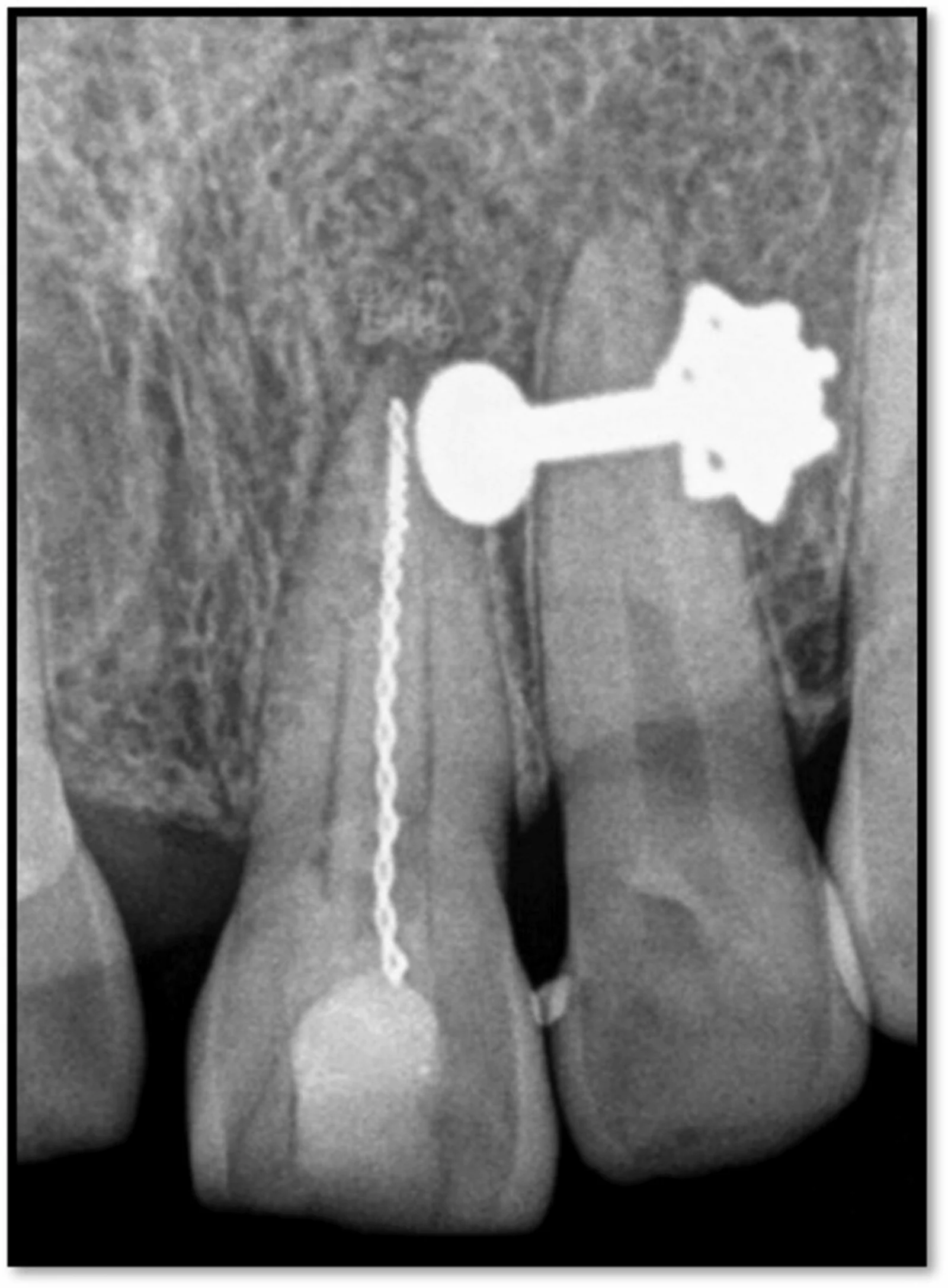
Case 1 follow-up showing complete resolution of periapical radiolucency

In Case 1, complete resolution of symptoms was reported at the one-week postoperative evaluation, with a VAS score of 0. The absence of symptoms was maintained throughout follow-up. Percussion and palpation remained negative, and no mobility or soft-tissue abnormalities were observed.

In Case 2, the preoperative pain score was a VAS score of 7. Pain decreased to a VAS score of 2 at one week and resolved to a VAS score of 0 at four weeks. Percussion sensitivity had resolved by the three-month follow-up. No post-obturation flare-up or other adverse event was reported in either case.

**Figure 14:**
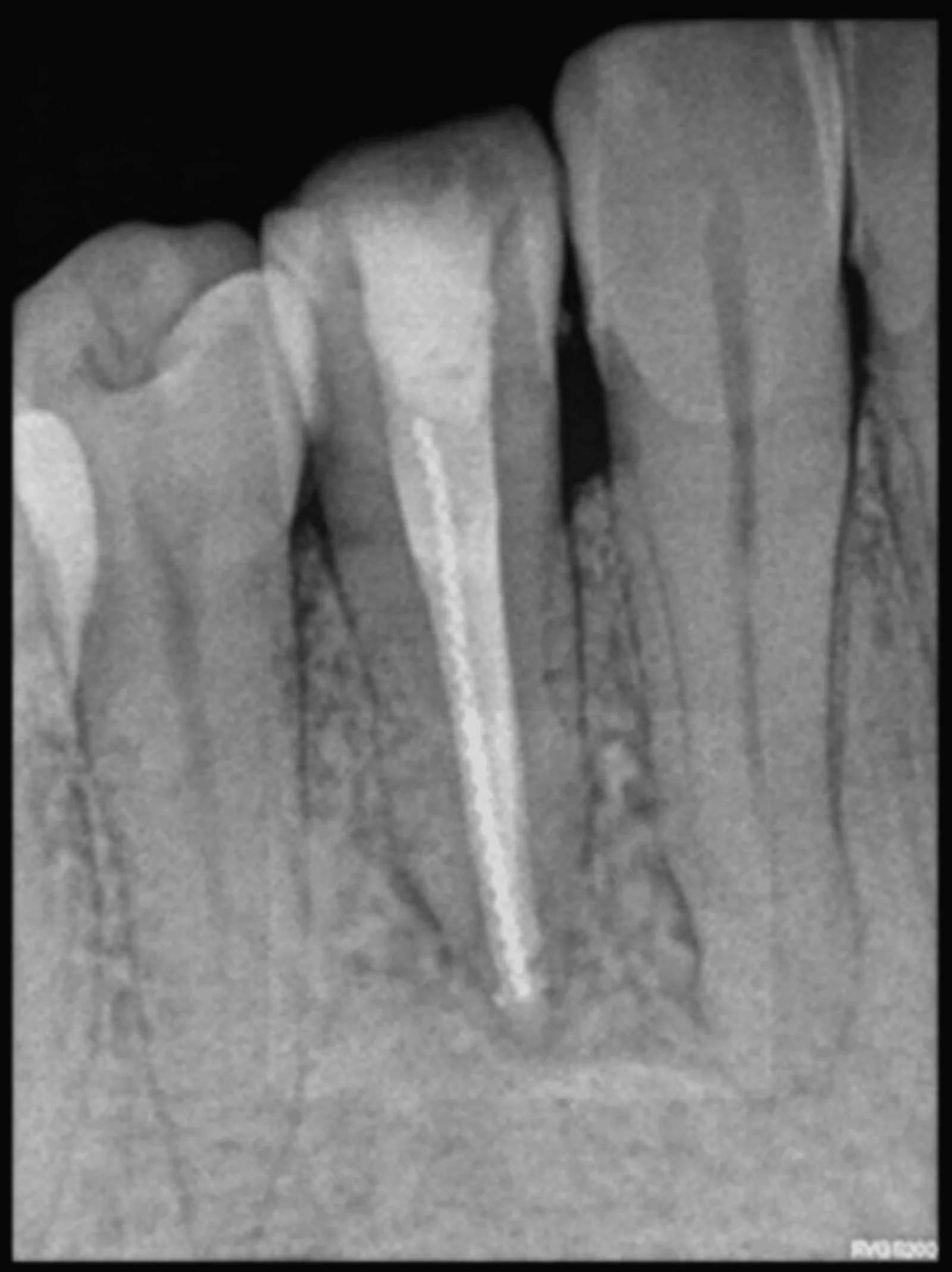
Case 2 follow-up radiograph showing complete periapical healing

Postoperative radiographs demonstrated adaptation of the custom-fit biomaterial to the canal anatomy in both cases. No evidence of biomaterial extrusion beyond the apex was observed.

At 24 months, both cases demonstrated complete radiographic resolution of the periapical radiolucency, with PAI scores improving from 4 preoperatively to 1 at the 24-month evaluation. No radiographic evidence of root resorption or secondary pathology was identified.

The definitive apical barrier placement was completed during a single definitive treatment visit in both cases. 

Proposed technical benefits and considerations

The proposed technique provides several potential technical advantages. First, the use of an intracanal impression permits fabrication of a plug based on the individual canal morphology rather than relying exclusively on freehand placement of biomaterial within the canal.

Second, fabrication outside the tooth permits direct visual inspection of the plug before clinical insertion. The split mold also facilitates retrieval of the partially set biomaterial without requiring manipulation deep within the root canal.

Third, the embedded twisted stainless-steel wire provides a simple carrier mechanism. The wire allows the operator to retrieve the plug from the mold and subsequently guide it into the prepared canal. The wire also provides structural support during manipulation. The present technique does not expose the wire directly to the oral environment; it remains surrounded by the set MTA/Biodentine. This encapsulation was intended to reduce direct exposure of the metallic surface to the oral environment. 

Fourth, the use of a predetermined plug configuration may facilitate controlled placement and may reduce the risk of unintended apical extrusion. In the two clinical applications described in this report, no extrusion was observed.

Finally, the technique was successfully applied using two different calcium-silicate biomaterials, Biodentine and MTA, suggesting that the concept may be adaptable to other moldable hydraulic biomaterials. However, this potential application requires independent validation.

Potential limitations of the technique

The technique also introduces additional procedural steps that are not required during conventional direct placement. Accurate recording of the canal anatomy is dependent on obtaining a complete and distortion-free impression. The impression must be retrieved without deformation and must adequately reproduce the apical region.

The extraoral fabrication step introduces additional handling of the biomaterial and requires careful control of the setting stage. Retrieval of the fabricated plug must be performed without fracture or deformation. The operator must also ensure that the embedded carrier remains securely incorporated within the biomaterial.

The applicability of the technique may be influenced by canal anatomy. Severely curved canals, narrow canals, or canals in which a complete intracanal impression cannot be obtained may present technical challenges.

The presence of a permanently embedded stainless-steel wire is another consideration. The present report does not provide long-term experimental evidence regarding the mechanical or biological behavior of the wire-biomaterial complex. Longer follow-up and laboratory investigations are therefore required.

A further limitation of this technical report is that three-dimensional adaptation and interfacial sealing of the customized apical biomaterial were not objectively evaluated using advanced imaging or microscopic methods. Therefore, the reported radiographic findings should be interpreted as confirmation of the position and apicocoronal extent of the customized plug rather than as evidence of complete three-dimensional adaptation or the absence of interfacial gaps.

## Discussion

The principal novelty of this technical report is the indirect fabrication of a patient-specific biomaterial plug using an intracanal elastomeric impression and a negative replica mold. Conventional apexification using calcium hydroxide may require prolonged treatment and multiple appointments, whereas calcium-silicate materials such as MTA and Biodentine permit more immediate establishment of an apical barrier [5,7]. Nevertheless, direct orthograde placement of a biomaterial in a wide-open apex remains dependent on operator control and may be complicated by extrusion or suboptimal barrier placement [8,9]. The comparative characteristics are presented in Table 1, which highlights the conceptual differences between conventional apexification approaches and the proposed carrier-supported custom-fit technique. 

**Table 1:** Comparative characteristics of conventional apexification approaches and the proposed carrier-supported custom-fit biomaterial technique

Feature	Calcium hydroxide apexification	Conventional direct MTA/Biodentine apexification	Proposed carrier-supported custom-fit technique
Apical barrier formation	Gradual [5]	Direct placement	Indirect prefabrication followed by placement
Biomaterial placement	Multiple intracanal applications [5,10]	Direct intracanal placement [6,7]	Custom-fit plug fabricated outside the canal
Anatomical customization	Limited	Operator dependent	Based on recorded canal morphology
Apical positioning	Variable	Operator dependent	Predetermined plug geometry with carrier-assisted placement
Risk of uncontrolled extrusion	Treatment dependent	Present [8,9]	Not established; no extrusion observed in the two reported cases.
Extraoral fabrication	No	No	Yes
Carrier mechanism	Not required	Not required	Embedded twisted stainless-steel wire
Definitive apical barrier procedure	Multiple treatment stages [10]	Usually completed in a limited number of visits	Completed in a single definitive placement visit in the two cases reported
Technical complexity	Moderate	Moderate	Higher
Current level of evidence	Established historical approach	Established clinical approach [6,7,8,9]	Preliminary; demonstrated in two clinical applications
Need for further validation	—	—	Yes

The technique described in the present report was developed to address this procedural challenge through extraoral fabrication of a custom-fit apical plug based on the recorded canal morphology. Unlike conventional direct placement, in which the clinician must manipulate the unset biomaterial within the canal and establish the desired dimensions intraoperatively, the proposed approach permits the apical component to be shaped before insertion. The canal impression records the internal morphology and transfers this information to a negative replica. The biomaterial can then be compacted into the replica under direct visual control. This concept may be particularly relevant where a wide or irregular apical opening makes conventional direct placement difficult.

A further feature of the technique is the embedded stainless-steel carrier. Previous approaches have described the use of internal matrices or other techniques to facilitate apical barrier placement [11]. In the present approach, the wire remains incorporated within the fabricated plug and serves as both a manipulation aid and a carrier during insertion. The wire was approximately 0.2 mm in diameter before twisting and approximately 0.5 mm after twisting. In the two cases reported here, it provided sufficient control to retrieve and place the custom-fit biomaterial plug.

The absence of biomaterial extrusion in both cases is encouraging. However, the present report cannot establish that the technique prevents extrusion. The predetermined dimensions of the plug may reduce the opportunity for uncontrolled extrusion, but the effect should be investigated experimentally using standardized models and larger clinical series.

The radiographic improvement observed in both cases was favorable. PAI scores decreased from 4 before treatment to 1 at 24 months [12]. Case 1 and Case 2 demonstrated progressive reduction in the periapical radiolucency. These observations are compatible with the reported clinical use of bioactive calcium-silicate materials in endodontic treatment [6,7]. Nevertheless, because only two clinical applications were performed and no control group was available, these outcomes should be interpreted as preliminary clinical observations rather than evidence of comparative effectiveness.

The biological activity of MTA and Biodentine has been associated with calcium ion release and the formation of calcium-phosphate or hydroxyapatite-like interfacial products [13-15]. The present technique does not alter the fundamental composition of either biomaterial; rather, it changes the manner in which the material is shaped and introduced into the canal. Therefore, the favorable clinical outcomes observed in these cases are biologically plausible, but the present report cannot directly establish the biological response at the material-tissue interface.

One potential advantage of the technique is the ability to inspect the fabricated plug before clinical placement. With conventional direct placement, the clinician must manipulate and condense the biomaterial within the canal while simultaneously controlling its apical position. In the proposed approach, the geometry of the plug is established before insertion. Radiographic verification can also be performed before and after placement, as demonstrated in the reported cases.

The technique may also reduce the amount of intraoperative manipulation required during definitive apical barrier placement. Although the definitive apexification step was completed in a single visit in both cases, the protocol still required a previous appointment for intracanal medication removal and impression acquisition. Quantitative comparisons of treatment time and radiation exposure should therefore be performed in future comparative studies.

Another consideration is the use of different biomaterials in the two cases. Biodentine was used in Case 1 and MTA in Case 2, demonstrating technical compatibility with both materials. However, because the materials were not compared under the same clinical or experimental conditions, the present report cannot determine whether one material is superior for this technique.

The principal limitations of the clinical evidence are the very small sample size, absence of a control group, and lack of laboratory validation. Only two clinical applications were reported, preventing statistical analysis and limiting generalizability. The 24-month follow-up provides encouraging clinical information but does not establish the long-term behavior of the embedded wire or the durability of the apical seal. Histological assessment was not performed, and therefore no direct conclusions can be made regarding tissue-level biological response.

Future studies should evaluate the technique using standardized extracted-tooth models before broader clinical application. Important parameters include dimensional accuracy of the fabricated plug, marginal adaptation, sealing ability, resistance to displacement, fracture behavior, the effect of the embedded wire on material properties, and the behavior of the biomaterial under simulated clinical conditions. Comparative clinical studies should subsequently assess treatment time, radiographic exposure, postoperative symptoms, apical healing, and long-term tooth survival.

## Conclusions

The principal significance of the proposed approach lies in its conceptual modification of apical barrier delivery rather than the introduction of a new biomaterial. The described technique provides an indirect method for fabricating and placing a custom-fit apical biomaterial plug in teeth with open apices. By combining an intracanal elastomeric impression, a negative replica mold, a hydraulic calcium-silicate biomaterial, and an embedded stainless-steel carrier, the technique allows the apical plug to be shaped before its definitive intracanal placement.

Clinical application in two teeth treated using Biodentine and mineral trioxide aggregate suggests the technical feasibility of the proposed approach and was associated with favorable clinical and radiographic outcomes over 24 months, including improvement in PAI scores from 4 to 1 and no observed biomaterial extrusion. These findings should be considered preliminary. Further laboratory and clinical studies are required to establish the dimensional accuracy, sealing ability, mechanical behavior, biological response, safety and biocompatibility of the permanently embedded stainless-steel carrier, and long-term clinical effectiveness of the proposed technique.
